# Naturally acquired functional antibody responses to group A *Streptococcus* differ between major strain types

**DOI:** 10.1128/msphere.00179-23

**Published:** 2023-09-20

**Authors:** Reuben McGregor, Aimee Paterson, Prachi Sharma, Tiffany Chen, Jarrod R. Lovell, Lauren H. Carlton, Andrew C. Steer, Joshua Osowicki, Jacelyn M. S. Loh, Nicole J. Moreland

**Affiliations:** 1 School of Medical Science, Faculty of Medical and Health Sciences, The University of Auckland, Auckland, New Zealand; 2 Maurice Wilkins Centre for Biodiscovery, The University of Auckland, Auckland, New Zealand; 3 Tropical Diseases Research Group, Murdoch Children’s Research Institute, Melbourne, Victoria, Australia; Duke Human Vaccine Institute, Durham, North Carolina, USA

**Keywords:** group A *Streptococcus*, M-type, immunity, opsonophagocytosis, antibody, human

## Abstract

Group A *Streptococcus* (GAS) is a leading human pathogen for which there is no licensed vaccine. Infections are most common in young children and the elderly suggesting immunity accumulates with exposure until immune senescence in older age. Though protection has been postulated to be strain type specific, based on the M-protein (*emm*-type), the antigenic basis of population-level immunity remains poorly understood. Naturally acquired GAS antibody responses were investigated using intravenous immunoglobulin (IVIG), which contains pooled immunoglobulins from thousands of healthy human donors, as a surrogate for population immunity. Functional opsonophagocytic killing assays were conducted with GAS strains (*n* = 6) representing the three major *emm*-pattern types (*emm*12, A-C pattern; *emm*53, D-pattern; and *emm*75, E-pattern). While IVIG induced opsonophagocytic killing of all GAS strains tested, specificity assays showed the profile of protective antibodies differed considerably between *emm*-types. Antibodies targeting the M-protein were a major component of the functional IVIG antibody response for *emm*12 and *emm*53 strains but not for *emm*75 strains. The striking differences in the contribution of M-protein specific antibodies to killing suggest naturally acquired immunity differs between strains from the major *emm-*patterns. This challenges the dogma that M-protein is the primary protective antigen across all GAS straintypes.

**IMPORTANCE:**

Group A *Streptococcus* (GAS) is a globally important pathogen. With the surge of invasive GAS infections that have occurred in multiple countries, contemporaneous with the relaxation of COVID-19 pandemic restrictions, there is increased interest in the mechanisms underpinning GAS immunity. We utilized intravenous immunoglobulin (IVIG), pooled immunoglobulins from thousands of healthy donors, as a surrogate for population-level immunity to GAS, and explored the contribution of strain-specific (M-type specific) antibodies to GAS immunity using functional killing assays. This revealed striking differences between major strain types as to the contribution of strain specific antibodies to killing. For GAS strains belonging to the E pattern group, M-type specific antibodies do not mediate killing and immunity, which contrasts with strains belonging to pattern A–C and D groups. This challenges the historical dogma, originally proposed by Rebecca Lancefield in the 1950–1960s, that the M-protein is the major protective antigen across all GAS strain types.

## OBSERVATION

*Streptococcus pyogenes* (group A *Streptococcus* [GAS]) is associated with a significant global burden of disease, across a diverse spectrum of clinical syndromes ranging from pharyngitis and impetigo to severe invasive infections and serious post-infectious sequelae such as acute rheumatic fever (1). A surge of invasive GAS infections in multiple countries has occurred contemporaneously with the relaxation of COVID-19 pandemic restrictions (2, 3). Immune protection against GAS is poorly understood. However, the epidemiological observation that GAS disease is most common among children and the elderly suggests that immunity accumulates through exposure until immune senescence in older age (1).

The GAS M-protein was first described as an immunodominant antigen and postulated as the major protective antigen by Rebecca Lancefield over 70 years ago. The hypervariable N-terminal region (HVR) of the protein is the basis of strain typing (*emm*-typing). Functional, *emm*-type-specific antibodies (where function is defined as the induction of phagocytosis) have been detected in humans decades after infection (4), and vaccination of animals with M-proteins (and related HVR peptide vaccines) can induce strong, protective immune responses (5). Structurally, M-proteins are elongated coiled coils with a gradient of sequence diversity from highly strain specific at the N-terminus (HVR) to very well conserved at the C-terminus (6). M-proteins vary in length depending on the extent of variable- and repeat-regions and have been categorized into a “three representative protein model” based on *emm* pattern type, with A–C patterns expressing the longest M-proteins, D-patterns intermediate, and E patterns the shortest (6). Most knowledge on *emm*-type specific immunity in humans comes from historical studies of A–C pattern strains. However, limited data for other strain types suggest that the relationship between *emm*-type and immunity is more complex (7).

**Fig 1:**
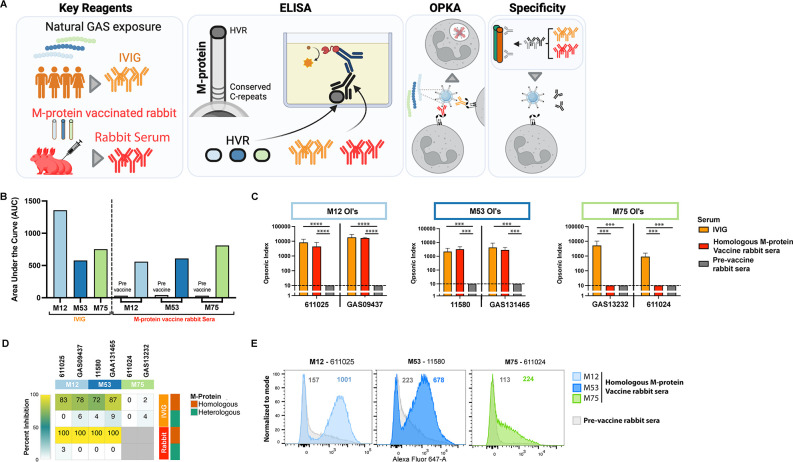
Characterization of IVIG and M-protein vaccine rabbit sera. (**A**) Study overview including key reagents used in the study (antibodies), M-type-specific HVR ELISAs used to characterize IVIG and sera (ELISA), opsonophagocytic killing assay showing antigen dependent killing of GAS (OPKA), and specificity assays showing pre-adsorption sera with M-proteins reducing killing in an OPKA (specificity). Colors are consistent with colors represented in panels B–E. Created with BioRender.com (**B**) ELISA detecting antibodies to M12 (light blue), M53 (dark blue), and M75 (green) HVR peptides in IVIG (orange), sera from rabbits vaccinated with whole M-protein (red), and their respective sera pre-vaccination (Pre vac, gray). Data represented as area under the curve (AUC) calculated in GraphPad Prism V9 (Fig. S2). (**C**) Opsonophagocytic killing assays showing opsonophagocytic killing capacity of IVIG, M-protein vaccinated rabbit serum, and respective rabbit serum pre-vaccination. Two strains of each M-type were tested, with strain names on the *x*-axis. Bars represent mean, and error bars represent standard deviation, *n* = 2. Dashed line represents the limit of detection of the OPKAs at an opsonic index of 10. A two-way ANOVA was carried out after the log10 transformation of opsonic indexes. ***P ≤ 0.001, ****P ≤ 0.0001 with Tukey correction for multiple comparisons. (**D**) Heat map showing percent inhibition of killing from pre-incubation of IVIG (orange) or rabbit serum (red) with either homologous (brown) or heterologous (green) M-protein (see Fig. S3). Data are mean percent inhibition from *n* = 2. Gray boxes represent no experiment conducted due to no killing observed against M75 strains with rabbit serum, as shown in panel **B**. Bar graphs representing the underlying data are included in Fig. S4. (**E**) Flow cytometry histograms showing binding of homologous M-protein vaccine rabbit sera to heat killed clinical GAS isolates. Control rabbit serum from respective rabbits’ pre-vaccination is shown as control in gray. Geometric mean fluorescence intensity is indicated in representative colors.

To explore the contribution of *emm*-type-specific antibodies to GAS immunity across patterns, we utilized optimized opsonophagocytic killing assays (OPKAs) (8) (Supplemental methods) in combination with intravenous immunoglobulin (IVIG) as a surrogate for population-level immunity (Fig. 1A). IVIG is a clinical blood product containing antibodies purified from thousands of healthy donors and has been similarly used to gain insight into *Streptococcus pneumoniae* immunity (9). The presence of GAS-specific antibodies in IVIG preparations is well documented, and it is used in the treatment of patients with invasive GAS infections (10). We investigated the functional antibody response for three different *emm*-types, each a prototype for the three major *emm*-patterns (*emm*12 for A-C pattern, *emm*53 for D-pattern, and *emm*75 for E-pattern) and included two clinical isolates for each (Table S1). The presence of *emm-*type-specific antibodies in IVIG (Privigen, CSL Behring) was confirmed using ELISA to 50-mer peptides derived from the HVR as well as the matched full-length recombinant M-proteins (Fig. S1) following published protocols (7) (Supplemental methods), with high titers observed for each of the three strain types (Fig. 1B; Fig. S2). The full-length recombinant M-proteins were shown to be folded with a high proportion of α-helical content by circular dichroism as expected for coiled-coil proteins (Fig. S1). Sera from rabbits vaccinated with the M-proteins were used as controls for type specific anti-M-antibodies as described (8) (Supplemental methods) and similarly showed high titers to homologous HVR peptides and M-proteins, whereas pre-vaccination showed no reactivity (Fig. 1B; Fig. S2).

IVIG enabled opsonophagocytic killing of every GAS isolate across the three *emm*-patterns (Fig. 1C). Homologous anti-M-protein rabbit sera also induced killing of both *emm*12 and *emm*53 isolates, yet neither of the *emm*75 isolates was killed by homologous sera in an OPKA (Fig. 1C). This suggests that the functional opsonophagocytic activity of M-protein targeted antibodies differ between strains. To dissect this further, specificity assays were undertaken by pre-adsorbing IVIG and rabbit sera with M-protein to deplete *emm*-type specific antibodies prior to the OPKA (Fig. S3). Complete (100%) inhibition of killing was achieved for *emm*12 and *emm*53 when rabbit sera were pre-adsorbed with homologous M-protein and very little or no inhibition was observed with heterologous M-protein pre-absorption (Fig. 1D; Fig. S4). This confirms that killing of *emm*12 and *emm*53 GAS by rabbit anti-sera was entirely due to M-protein specific antibodies, as expected. As killing of *emm*75 isolates was not observed with matched rabbit sera, specificity assays could not be conducted. High inhibition of killing was also observed for both *emm*12 (80.5%) and *emm*53 (79.5%) when IVIG was pre-adsorbed with homologous M-proteins, and little or no inhibition was observed with heterologous M-protein pre-adsorption (Fig. 1D; Fig. S4). Thus, functional antibodies in IVIG, which induce *emm*12 and *emm*53 killing, were predominantly M-protein specific. In contrast, IVIG induced-killing of *emm*75 strains was not inhibited by M75 protein pre-adsorption (Fig. 1D; Fig. S4), suggesting that killing of the *emm*75 strains was not M-protein mediated.

The absence of functional, *emm*-type-specific antibody mediated killing in both matched rabbit sera and IVIG against *emm*75 compared with *emm*12 and *emm*53 highlights distinct differences between the pattern E and the pattern A–C and D strains investigated. This may be driven by differences in the size as well as the functional and structural characteristics of the M-proteins on pattern A–C and D strains, compared with M-proteins expressed by pattern E strains (6, 11). Flow cytometry was performed with anti-M-protein rabbit serum and one representative clinical GAS isolate per *emm-*pattern-type. This included an Fc blocking step to reduce non-specific interactions (Supplemental methods) and showed markedly reduced binding of matched anti-M-protein sera to *emm*75 compared with *emm*12 and *emm*53 (Fig. 1E). This suggests reduced accessibility of anti-M antibodies to the shorter, pattern E M-proteins on the surface of the GAS bacterium, or alternatively, reduced M-protein expression in these strain types. Either explanation has the same functional consequence; population-level immunity, as represented by functional antibody responses in IVIG, does not appear to be M-protein mediated for *emm*75 strains in contrast to strains from the other major *emm-*pattern types examined (A–C and D). The antigenic basis of *emm*75 killing remains to be determined, but the recently developed *emm*75 human challenge model provides a novel means for further investigation (12, 13).

The lack of M-protein mediated killing of the *emm*75 E pattern strains in this study challenges the dogma that M-protein is the major protective antigen across all GAS strain types. This study was limited by the number of strains investigated. The findings may not be reflective of all pattern E strains, but the findings nevertheless suggest that naturally acquired, protective antibodies found in IVIG target alternative antigens on some GAS strains. This does not preclude the possibility that future vaccines, based on either type-specific or conserved vaccine antigens, will be broadly protective across the spectrum of GAS strains. There are likely to be significant differences between infection- and vaccine-acquired immunity, as has been shown for other vaccine preventable diseases, including *S. pneumoniae*(9). Overall, these findings provide important insight into the biological basis of naturally acquired immunity for GAS and highlight the importance of strain selection when investigating correlates of protection.

## SUPPLEMENTAL MATERIAL

Supplemental material msphere.00179-23-s0001.docx Supplemental methods, Table S1, and Figures S1 to S4.

ASM does not own the copyrights to Supplemental Material that may be linked to, or accessed through, an article. The authors have granted ASM a non-exclusive, world-wide license to publish the Supplemental Material files. Please contact the corresponding author directly for reuse.
