# Supplementary material for: Naturally acquired functional antibody responses to group A Streptococcus differ between major strain types
Source: mSphere. 2023 Sep 20;8(5):e00179-23. doi: 10.1128/msphere.00179-23 (PMC10597462; doi:10.1128/msphere.00179-23)
Supplement: Supplemental material — Supplemental methods, Table S1, and Figures S1 to S4. [file msphere.00179-23-s0001.docx]

# Supplementary material

## Supplementary Methods

### Protein Production and Rabbit Antisera

M-proteins and rabbit anti-M-protein sera were generated as previously described ^1^. For M53, the *emm*53 gene (GAS131465) was amplified from genomic DNA and cloned into the pET32a3c expression vector and the sequence was confirmed by Sanger sequencing. For M12 and M75, the sequence encoding the respective *emm* genes (*emm*12 (HKU16) and *emm*75 (611024)) were synthesised and cloned into pET151/D-TOPO (Thermo Fisher Scientific). Each M-protein was expressed in BL21(DE3) pLysS *E. coli* with the signal sequence and transmembrane regions removed and an N-terminal His_6_-tag. Purification involved immobilized metal (Ni-NTA) affinity chromatography, subsequent His_6_-tag cleavage with 3C (M53) or rTEV (M12 and M75) protease, and a final purification step of gel filtration. Proteins were determined to be >95% pure by SDS‑PAGE (**Suppl. Fig. 1A**).

Circular dichroism (CD) was utilized to assess the degree of secondary structure of the recombinant M-proteins. Far UV spectra (180-320nm, 0.5nm steps) was recorded using a Chirascan CD spectrometer (Applied Photophysics) at 16°C for five iterations and averaged. Baseline absorbance of the buffer alone was subtracted and spectra were smoothed following conversion to mean residue ellipticity [θ] in deg cm2 decimol-1 using the equation [θ] = 100∆θ/Cnl where ∆θ is the difference in millidegrees between the protein sample and buffer, C is protein concentration (mM), n is the number of residues in the protein and l is the cuvette path length (cm) (**Suppl. Fig. 1B**).

To generate antisera, female New Zealand white rabbits were immunized subcutaneously with 200µg of M-protein in Incomplete Freund’s adjuvant (Sigma). Immunization was performed at 0, 2 and 4 weeks followed by exsanguination at week 6.

### HVR ELISA

Enzyme-linked immunosorbent assays (ELISAs) were conducted with synthetic peptides derived from the N-terminal 50 amino acids of the respective M-proteins as published^2^. The peptides were synthesized commercially (GenScript) to >90% purity. Plates were coated with 5 µg/mL M-peptide HVR for 3 hours 37°C followed by overnight at 4°C. Rabbit sera and IVIG were serially diluted 3-fold from a starting dilution of 1:1000 and incubated for 2 hours at 37°C followed by addition of an HRP-labelled anti-human IgG (Abcam) for IVIG and anti-rabbit IgG (Abcam) for rabbit anti-sera as detection. Optical density was measured at 450nm using an EnSpire plate reader plate reader (Perkin Elmer).

### Osonophagocytic Killing Assays

Opsonophagocytic killing assays (OPKAs) were carried out as previously described^1,3^. In brief, HL-60 cells were differentiated by incubation with 0.8% dimethylformamide (DMF) at 37°C with 5% CO_2_ for 3 or 4 days and diluted in opsonization buffer to 1 × 10^7^ cells/ml. Frozen bacterial stocks were washed and diluted in opsonization buffer (10% v/v heat-inactivated FBS (HyClone)), 0.1% w/v gelatin (Sigma) in Hanks’ balanced salt solution (HBSS) with Ca/Mg) to ~120,000 CFU/ml and incubated for 30 min at room temperature, at 700 rpm, with 20 µl heat-inactivated sera or IVIG serially diluted. Ten microliters of pre-diluted baby rabbit complement and 40 µl of differentiated human HL-60 cells were added and incubated for 60 minutes at 37°C with 5% CO_2_, at 700 rpm. Plates were then incubated on ice for 20 min, and 10 µl from each well was spotted onto Todd-Hewitt-Yeast (THY) agar plates. An overlay agar (THY, 0.75% w/v bacteriological agar, and 0.005% 2,3,5-tetraphenyltetrazolium chloride (Sigma)) was poured onto each plate. Plates were incubated overnight at 37°C with 5% CO_2_. The surviving bacteria (CFU) was enumerated using a ProtoCOL3 automated colony counter (Synbiosis). The dilution of sera resulting in 50% killing was calculated as the opsonic index (OI).

Specificity assays were carried out as above except that IVIG and rabbit serum were pre‑adsorbed for 30 minutes at room temperature with 20μg/mL and 100μg/mL of M‑protein, respectively, before addition into the assay. These concentrations of M-protein equated to the maximum inhibition achievable in the sera/IVIG following a titration of M‑protein. The percent (%) inhibition was calculated as: CFU[antisera:antigen]-CFU[0% inhibition control])/(CFU [max killing control]-CFU[0% inhibition control]) *100 (**Suppl. Fig.3**).

### Flow cytometry

GAS strains were thawed at 37℃, re-suspended in buffer (3% v/v heat inactivated Fetal Bovine Serum (FBS) (Hyclone) 5mM EDTA) to 0.2 Optical density (OD at 600nM) and heat killed at 80℃ for 5 minutes. Following sonication, bacteria were washed and resuspended to contain 1,000,000 cells per 200 µl FACS buffer (1% v/v heat inactivated FBS, 5mM EDTA). Samples were then spun, supernatant removed, and blocked with 100µl of Human Antibody Fc region (AbCam) (1:20) for 10 minutes at room temperature, stained with 100µl of rabbit sera (1:50) and incubated on ice for 30 minutes. Samples were washed using FACs buffer and incubated with 100µl of anti-rabbit Alexafluor 647 (Life Technologies) for 30 minutes on ice. Samples were washed and re-suspended in 200µl FACs buffer and run on a BD LSRII flow cytometer (BD Sciences) and analyzed using FlowJo (v10).

## Supplementary Tables and Figures

**Supplementary Table 1. |Group A *Streptococcus* Isolates used in this study|** Isolate characteristics including *emm*‑type, *emm-*pattern, the site of original isolation, clinical syndrome, and accession number (either whole-genome sequence or other sequence deposition from the same isolate).

| ***emm* type**/Isolate | *emm-*pattern | Source | Disease Manifestation | Accession number | Country of origin |
| --- | --- | --- | --- | --- | --- |
| ***emm*12** | A-C |  |  |  |  |
| 611025 |  | Throat | Pharyngitis | SRR8217179 | Australia |
| GAS09437 |  | Throat | ARF | GCA_900994325 | New Zealand |
| ***emm*53** | D |  |  |  |  |
| 11580 |  | Throat | Pharyngitis | KJ816974 | New Zealand |
| GAS131465 |  | Throat | ARF | GCA_900984045 | New Zealand |
| ***emm*75** | E |  |  |  |  |
| GAS13232* |  | Throat | Pharyngitis | / | New Zealand |
| 611024 |  | Throat | Pharyngitis | CP033621 | Australia |

**emm*-typing previously conducted using standard protocols

**Supplementary Figure 1 |Characterization of the recombinant M-proteins| (A)** Reducing SDS‑PAGE of the three recombinant M-proteins. The predicted monomeric molecular weights are 56.88 kDa (M12), 36.12 kDa (M75) and 40.53 kDa (M53). **(B)** Circular dichroism spectra of the recombinant M-proteins (rM). Each spectra shows the characteristic double minimum at 208nm and 222nm that is typical of α-helical proteins^4^.

**
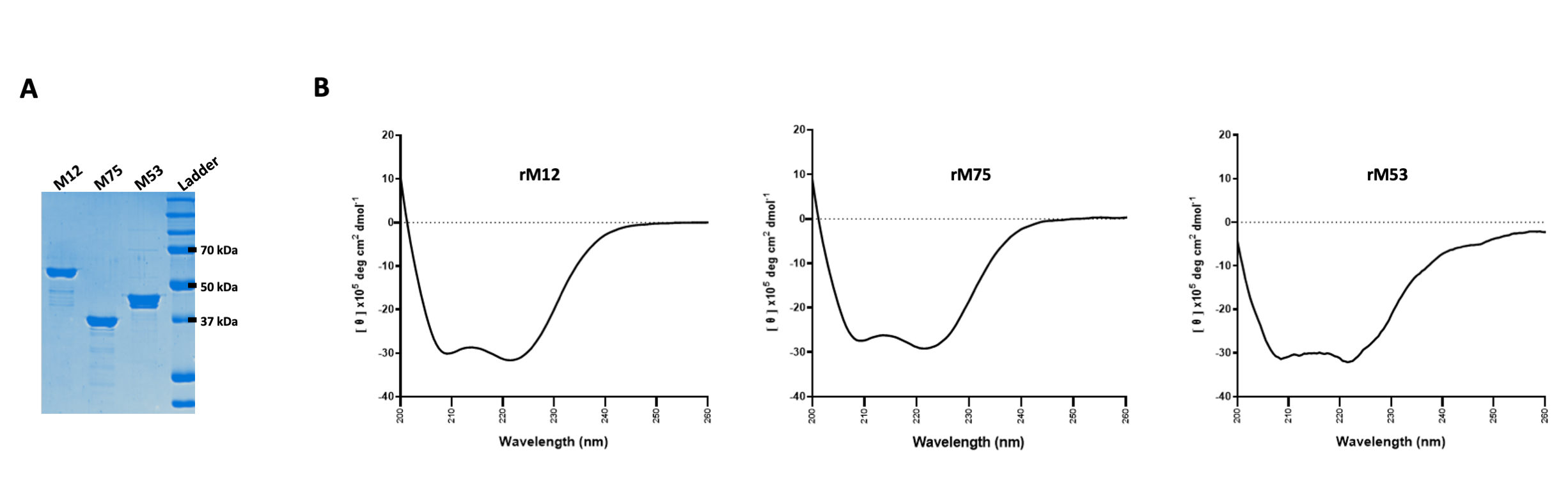
**

**Supplementary Figure 2 |HVR ELISA titration curves|** IVIG (orange; **B**,**D** and **F**) and serum from rabbits vaccinated with M-protein (homologous Rb; **A**, **C** and **E**) or serum from the same rabbit’s pre-vaccination (white) were serially diluted 7-fold and added to wells coated with 50-mer HVR peptides (circles) or full length (FL) M-protein (squares); M12 (light blue), M53 (dark blue) or M75 (green). The area under each curve was then calculated as shown in **A** for each FL M-protein and HVR (grey shaded area’s) using GraphPad Prism V9. (**G**) In order to characterize the specificity of the rabbit sera further, each rabbit sera were run against all three HVRs, showing each rabbit sera only reacted to its homologous HVR.

**Supplementary Figure 3 |Specificity assays and calculation|**. Representative specificity assay for M12: 611025 GAS and IVIG. **(A)** An OPKA (line graph) was performed to determine the serum dilution at which maximum killing occurs (represented by double headed black arrow). The solids horizontal line indicates the maximum average growth, and the dashed line indicates the accepted 50% titer point for a standard OPKA. A specificity assay at the designated max killing serum dilution was conducted by pre-adsorbing sera at this dilution with either homologous (brown, in this case M12 protein) or heterologous (green) M-protein and subsequent OPKA steps were as normal. **(B)** The relevant CFU’s for percent (%) inhibition calculation (as described in supplementary methods) from n=2 experiments are shown, with lines joining individual experiments and bar representing the median. The % Inhibition for the respective GAS/sera combinations were then used to plot the heat-map in **Fig.1**.

**Supplementary Figure 4 |Specificity Assay Bar Graphs|** Percent inhibition of killing from pre-incubation of IVIG **(A)** or rabbit serum **(B)** with either homologous (brown) or heterologous (green) M-protein. Bars represent mean and error bars standard error of the mean from n=2. An unpaired t-test was carried out with ** P ≤ 0.01 *** P ≤ 0.001, **** P ≤ 0.0001 with Tukey correction for multiple comparisons.


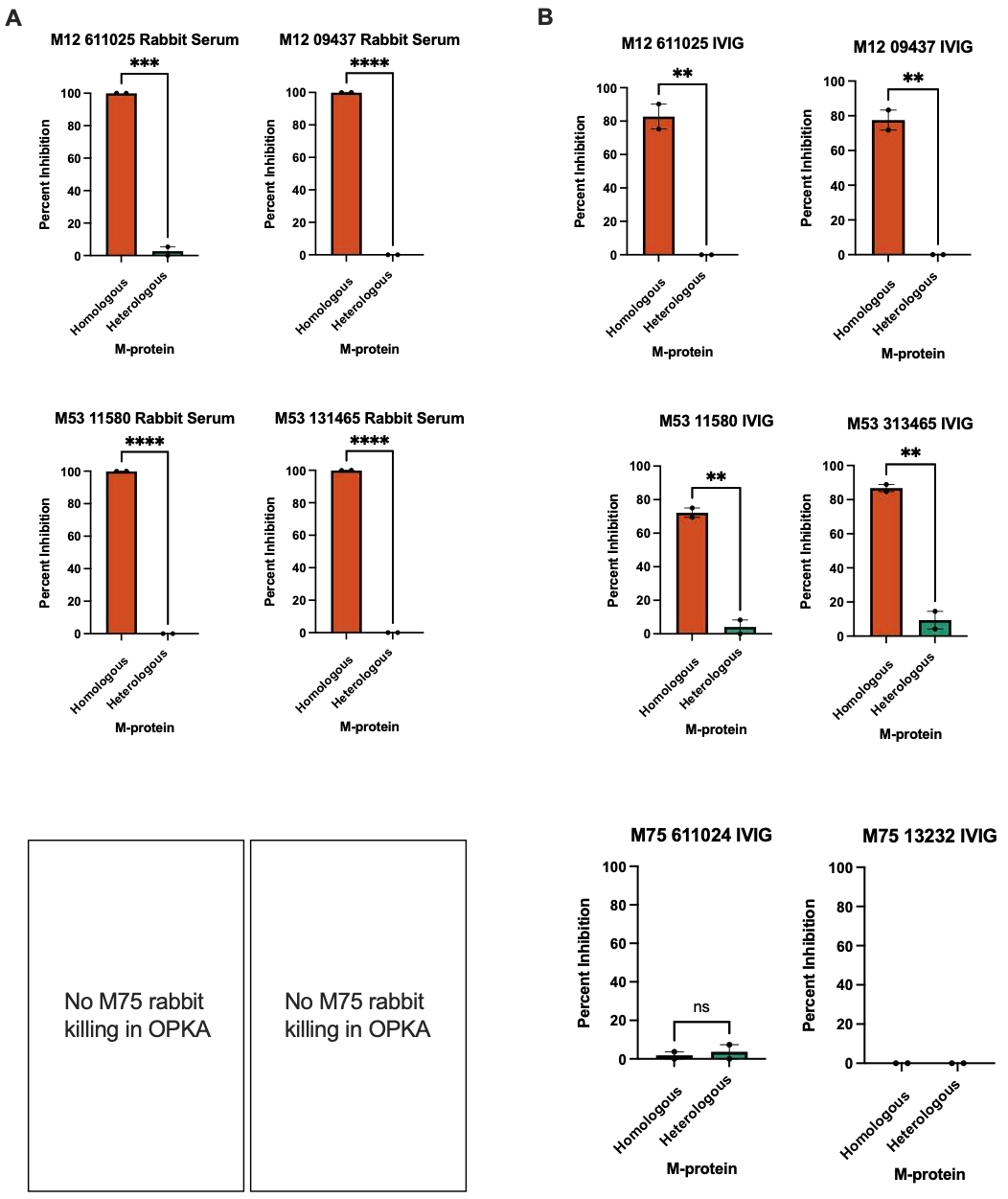


## Supplemental references:

1 Jones S, Moreland NJ, Zancolli M, Raynes J, Loh JMS, Smeesters PR *et al.* Development of an opsonophagocytic killing assay for group a streptococcus. *Vaccine* 2018; 36: 3756–3763.

2 Frost HR, Laho D, Sanderson-Smith ML, Licciardi P, Donath S, Curtis N *et al.* Immune Cross-Opsonization Withinemm Clusters Following Group AStreptococcus Skin Infection: Broadening the Scope of Type-Specific Immunity. *Clin Infect Dis* 2017; 65: 1523–1531.

3 McGregor R, Jones S, Jeremy RM, Goldblatt D, Moreland NJ. An Opsonophagocytic Killing Assay for the Evaluation of Group A Streptococcus Vaccine Antisera. *Methods Mol Biology Clifton N J* 2020; 2136: 323–335.

4 Wei Y, Thyparambil AA, Latour RA. Protein helical structure determination using CD spectroscopy for solutions with strong background absorbance from 190 to 230nm. *Biochim Biophys Acta (BBA) - Proteins Proteom* 2014; 1844: 2331–2337.
